# The Five *AhMTP1* Zinc Transporters Undergo Different Evolutionary Fates towards Adaptive Evolution to Zinc Tolerance in *Arabidopsis halleri*

**DOI:** 10.1371/journal.pgen.1000911

**Published:** 2010-04-15

**Authors:** Zaigham Shahzad, Françoise Gosti, Hélène Frérot, Eric Lacombe, Nancy Roosens, Pierre Saumitou-Laprade, Pierre Berthomieu

**Affiliations:** 1 Biochimie et Physiologie Moléculaire des Plantes, Montpellier SupAgro, CNRS – INRA – Université Montpellier II, Montpellier, France; 2 Laboratoire de Génétique et Evolution des Populations Végétales, CNRS – Université des Sciences et Technologies de Lille, Villeneuve d'Ascq, France; University of Georgia, United States of America

## Abstract

Gene duplication is a major mechanism facilitating adaptation to changing environments. From recent genomic analyses, the acquisition of zinc hypertolerance and hyperaccumulation characters discriminating *Arabidopsis halleri* from its zinc sensitive/non-accumulator closest relatives *Arabidopsis lyrata* and *Arabidopsis thaliana* was proposed to rely on duplication of genes controlling zinc transport or zinc tolerance. *Metal Tolerance Protein 1* (*MTP1*) is one of these genes. It encodes a Zn^2+^/H^+^ antiporter involved in cytoplasmic zinc detoxification and thus in zinc tolerance. *MTP1* was proposed to be triplicated in *A. halleri*, while it is present in single copy in *A. thaliana* and *A. lyrata*. Two of the three *AhMTP1* paralogues were shown to co-segregate with zinc tolerance in a BC1 progeny from a cross between *A. halleri* and *A. lyrata*. In this work, the *MTP1* family was characterized at both the genomic and functional levels in *A. halleri*. Five *MTP1* paralogues were found to be present in *A. halleri*, *AhMTP1*-*A1*, -*A2*, -*B*, -*C*, and -*D*. Interestingly, one of the two newly identified *AhMTP1* paralogues was not fixed at least in one *A. halleri* population. All *MTP1s* were expressed, but transcript accumulation of the paralogues co-segregating with zinc tolerance in the *A. halleri* X *A. lyrata* BC1 progeny was markedly higher than that of the other paralogues. All *MTP1s* displayed the ability to functionally complement a *Saccharomyces cerevisiæ* zinc hypersensitive mutant. However, the paralogue showing the least complementation of the yeast mutant phenotype was one of the paralogues co-segregating with zinc tolerance. From our results, the hypothesis that pentaplication of *MTP1* could be a major basis of the zinc tolerance character in *A. halleri* is strongly counter-balanced by the fact that members of the *MTP1* family are likely to experience different evolutionary fates, some of which not concurring to increase zinc tolerance.

## Author Summary

*Arabidopsis halleri* has developed the characters of zinc hypertolerance and hyperaccumulation as compared to its close relatives *Arabidopsis thaliana* and *Arabidopsis lyrata*. Different candidate genes were proposed to account for the appearance of these characters in *A. halleri*. One of them is *MTP1* (*Metal Tolerance Protein 1*), which is involved in cytoplasmic zinc detoxification. We found that *A. halleri* harbored five gene copies of *MTP1*, whereas *A. thaliana* and *A. lyrata* possess a single copy. It is thus tempting to associate the zinc hypertolerance character of *A. halleri* to the pentaplication of *MTP1*. However, we observed that one of the five *MTP1* copies is not fixed in a population growing on metal contaminated soil. Also, the different *MTP1* genes are markedly differentially expressed in *A. halleri*, two of them being poorly expressed and repressed in response to zinc constraint. *AhMTP1* copies were also demonstrated to display a differential ability to complement the zinc hypersensitivity of a yeast mutant that is dysfunctional in vacuolar zinc transporters. Our findings suggest that different evolutionary fates, some of them not concurring to increase zinc tolerance, are likely to take place for the members of the *MTP1* family in *A. halleri*.

## Introduction

Adaptation of an organism to a challenging environment entails dramatic modifications in cellular, physiological, and regulatory processes. Gene duplications are postulated to be one of the main mechanisms providing raw genetic material for the origin of these adaptive modifications [1]. Conversely, the absence of gene duplications is thought to severely limit the plasticity for a genome or species in adapting to a challenging environment [2]. Plants are particular in the regard that they exhibit higher percentage of duplicated genes than other organisms [2]. Within the Plant kingdom, comparative genomic studies unravelled lineage-specific differential expansion of gene families in different plants species [3]–[5]. Much of the plant diversity may have arisen following the duplication and adaptive specialization of pre-existing genes rather than following the invention of new gene(s) [6]. After duplication and once fixed within species, three possible fates are typically envisaged for duplicated genes/paralogues [7]. Because selective constraints can be relaxed on duplicated genes initially underlying a same function, degenerative mutations can occur and result in the loss of function for one of the gene copies, therefore creating a pseudogene (non-functionalization). Alternatively, a new advantageous mutation can occur and confer a new function to one of the gene copy (neo-functionalization). Finally, rather than one gene duplicate retaining the original function and the others either degrading or evolving new functions, the original function of the single-copy gene may be partitioned among the duplicates (sub-functionalization). Thus, whereas orthologues in different species are usually expected to share similar functions, paralogues within a genome could have no or different functions.

Studying plant adaptation to extreme environments such as metal contaminated areas is an excellent way to characterise mechanisms underlying the evolution of a species, especially since there are only very few species that can survive and reproduce in these extreme environments [8]. In addition, metal tolerance is an important trait to investigate because its understanding can help to develop phytoremediation strategies for polluted soils [9]. Analysis of the natural adaptive evolution towards zinc hypertolerance in plants has attracted the attention of the scientific community due to the availability of diverse flora. Indeed, the same genus can host species showing zinc hypertolerance together with species showing zinc sensitivity. This is the case in the Arabidopsis genus: *Arabidopsis halleri* is a zinc hypertolerant species while its two closest relatives *Arabidopsis thaliana* and *Arabidopsis lyrata* are zinc sensitive. For example, *A. halleri* seedlings grown on 500 µM zinc appeared healthy and showed continued growth while *A. thaliana* and *A. lyrata* seedlings displayed dry and chlorotic leaves and stopped growing in presence of 100 µM zinc in the culture medium [10]. Zinc tolerance, together with zinc hyperaccumulation, are constitutive traits in *A. halleri* [11]. These traits have been acquired quite recently in *A. halleri*, since this species is estimated to have diverged from *A. lyrata* and *A. thaliana* ∼2 and ∼5 million years ago, respectively [12]. A significant level of quantitative variation of zinc tolerance was however observed among *A. halleri* populations [10], [11]. In particular, populations collected on metalliferous soils (known as metallicolous populations) display enhanced zinc tolerance compared to populations collected in metal uncontaminated sites (known as non metallicolous populations) [11]. Interestingly, a phylogeographic survey proposed that geographically distant metallicolous populations have been founded independently in distinct polluted areas [13] suggesting that the adaptive improvement of zinc tolerance may involve distinct genetic mechanisms in distinct metallicolous populations.

As already mentioned, *A. halleri* has experienced dramatic alterations of mechanisms involved in zinc tolerance. Gene duplications were proposed to underlie acquisition of this character in *A. halleri* [14], [15]. For instance *ZIP3*, *ZIP6* and *ZIP9*, members of the zinc-regulated transporter/iron-regulated transporter-like proteins family [16], the *HMA4* transporter controlling root to shoot zinc transport and zinc tolerance [15] and the *MTP1* transporter controlling zinc transport into the vacuole [17] were shown to display increase in gene copy number in *A. halleri* compared to in *A. thaliana* or in *A. lyrata*. MTP1 (METAL TOLERANCE PROTEIN 1) is a Zn^2+^/H^+^ antiporter effluxing zinc out of the cytoplasm [17], [18]. When ectopically over-expressed in *A. thaliana*, AtMTP1 confers enhanced zinc tolerance [19]. *MTP1* is present as single copy in *A. thaliana*. In *A. halleri*, three orthologues to the unique *A. thaliana MTP1* were reported: *AhMTP1-A*, *AhMTP1-B* and *AhMTP1-C* [14]. Altogether, these *AhMTP1s* are over-expressed in *A. halleri* as compared to in *A. thaliana*. From the analysis of a backcross between the zinc hypertolerant *A. halleri* spp. *halleri* and the zinc sensitive *A. lyrata* spp. *petraea*, the three *AhMTP1* paralogues were mapped to 3 different linkage groups. Two of the three paralogues, *AhMTP1-A* and *AhMTP1-B*, co-segregated with zinc tolerance QTLs controlling short-term root elongation in response to zinc constraint, while *AhMTP1-C* did not [20].

Until now detailed characterisation had not been performed for each of the *AhMTP1* orthologues. This was a limiting step both for the further functional characterization of this gene sub-family in *A. halleri* and for the analysis of the genetic evolution of this family in relation to adaptation to zinc in *A. halleri*. In this work, cloning, sequencing and genetic mapping of all the *A. halleri MTP1* paralogues revealed two new *MTP1* paralogues. *A. halleri* was thus found to harbour five *AhMTP1* paralogues, *AhMTP1*-*A1*, -*A2*, -*B*, -*C* and -*D*. The *AhMTP1*-*D* paralogue is not fixed in the metallicolous Auby population. Transcript accumulation studies together with functional characterizations in a heterologous yeast system enabled to propose that the different *MTP1* duplications in *A. halleri* had different evolutionary fates including non-functionalization, sub-functionalization, and neo-functionalization. These results should provide a strong basis for further genetic diversity and linkage disequilibrium studies in *A. halleri*.

## Results

### Identification of all the *MTP1* paralogues in *A. halleri*

In order to identify all the members of the *MTP1* family in *A. halleri*, a BAC library [21] was screened with a labelled full length *AhMTP1* probe obtained by PCR using primers designed from the published *A. halleri MTP1* mRNA sequence (AJ556183 accession). Eight BAC clones, 3F23, 7G24, 16A6, 12L21, 1O21, 2B14, 3I23 and 1F18 were identified and confirmed by PCR sub-screening using the same primers.

**Figure 1:**
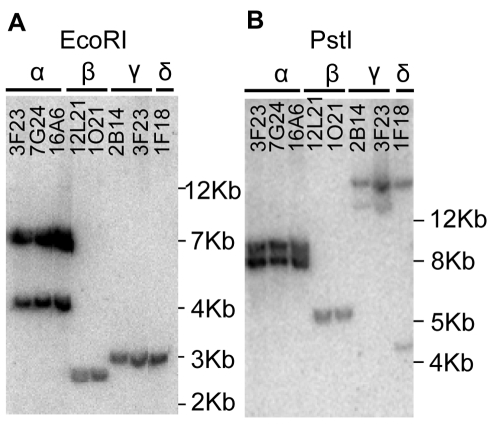
Southern hybridization of *A. halleri MTP1* harbouring BAC clones. BAC DNA digested with *Eco*RI (panel A) or *Pst*I (panel B) was probed with a full length *AhMTP1* probe. Names of the BAC clones are given at the top of the lanes. The α, β, γ, and δ symbols are the names given to the four distinct hybridisation profiles. Sizes of ladder are shown on the right of each panel.

*A. halleri* was initially proposed to harbour three *MTP1* paralogues [14]. With these data in mind, grouping of the eight BAC clones was attempted from the Southern hybridization profiles obtained using four different restriction enzymes, *Eco*RI, *Hin*dIII, *Nco*I and *Pst*I (Figure 1 and data not shown). The eight BAC clones could be arranged into four groups, α, β, γ and δ displaying different profiles. γ and δ groups displayed however closely related profiles: their *Eco*RI profile was identical (Figure 1A) and their profile for other restriction enzymes shared a common band (Figure 1B and data not shown). Since *A. halleri* is an out-crossing species, BAC clones from groups γ and δ might harbour two allelic forms of a single locus. Alternatively, they may represent two similar but distinct loci harbouring *AhMTP1* paralogues. These analyses thus suggested that *A. halleri* could harbour more than three *MTP1* loci.

### Genetic mapping revealed the existence of a new *MTP1* locus in *A. halleri*

**Figure 2:**
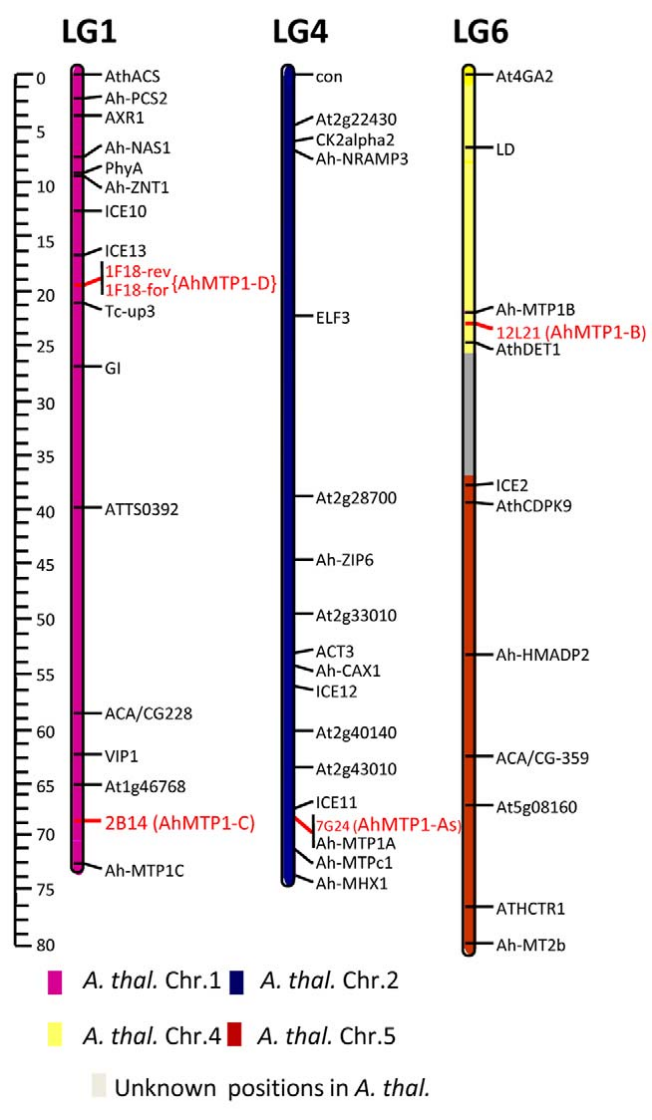
Mapping of the *AhMTP1* paralogues on the *A. halleri* × *A. lyrata petraea* BC1 linkage map. The mapping was performed from the analysis of 199 plants and the four parents of an *A. halleri* × *A. lyrata petraea* BC1 population. Positions of BAC clones 7G24, 12L21, 2B14, and 1F18 that harbour the *AhMTP1*-*A1* & -*A2*, *AhMTP1*-*B*, *AhMTP1*-*C*, and *AhMTP1*-*D* paralogues, respectively, are indicated in red letters (1F18rev and 1F18for are two independent markers). The other markers presented on the map have been previously described [20]. Only linkage groups (LG) 1, 4, and 6 of the *A. halleri* genome are shown. Colours of the bars representing the three linkage groups refer to the conserved synteny between the *A. halleri* and the *A. thaliana* genomes as inferred [22]. The regions showing no conserved synteny with *A. thaliana* are indicated in grey colour. The map was constructed using Joinmap 3.0. The scale to the left of figure represents centi-morgan distances.

The three already described *A. halleri MTP1* paralogues, *AhMTP1-A*, *AhMTP1-B* and *AhMTP1-C,* had been mapped to the bottom of linkage group 4, to the top of linkage group 6 and to the bottom of linkage group 1 on the *A. halleri* X *A. lyrata* linkage map, respectively [20]. To associate the 4 groups of BAC clones that we identified with the already described *MTP1* paralogues, genetic mapping was performed using markers derived from selected BAC clones representing each group (Figure 2). The mapped positions of BAC clones 7G24, 12L21 and 2B14 representing the α, β, and γ groups, respectively, corresponded to the already mapped positions of *AhMTP1-A*, *-B* and *-C*, respectively. Thus the *MTP1* copies characterising the α, β, and γ groups were considered to correspond to the *AhMTP1-A*, *AhMTP1-B* and *AhMTP1-C* paralogues, respectively. The BAC clone 1F18, which represents the δ group, was mapped to the upper part of linkage group 1 on the *A. halleri* X *A. lyrata* linkage map. The positioning was ascertained using 2 independent markers derived from each end of the BAC clone (Figure 2). No known *MTP1* paralogue had already been mapped at that locus. Therefore, the *MTP1* copy characterising the δ group was considered as a new *A. halleri MTP1* paralogue and was named as *AhMTP1-D*.

The unique *AtMTP1* gene (At2g46800) is located at the bottom of chromosome II of *A. thaliana*. Considering the shared synteny between the genome of *A. thaliana* and the genomes of other brassicaceae [22], position of the *AtMTP1* locus corresponds to the position of the *AhMTP1*-*A* locus (Figure 2).

### Sequence analyses unravel five *AhMTP1* paralogues showing a significant diversity in non-coding regions

**Figure 3:**
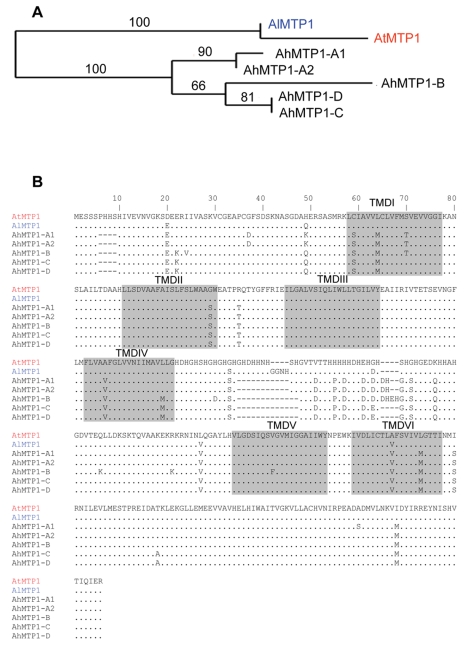
Phylogenetic analysis of *MTP1* gene sub-family from *A. thaliana*, *A. lyrata*, and *A. halleri*. (A) Maximum likelihood tree of MTP1 protein sequences from *A. thaliana*, *A. lyrata* and *A. halleri*. Bootstrap values are indicated in percentage (100 replicates). (B) Alignment of predicted amino acid sequences of *MTP1s* from *A. thaliana*, *A. lyrata* and *A. halleri*. Sequences are represented in 80 amino acids long blocks. The AtMTP1 protein sequence is from accession NP_850459 and the AlMTP1 one was extracted from scaffold 4 of the *A. lyrata* sequencing project available on http://genome.jgi-psf.org/cgi-bin/runAlignment?db=Araly1&advanced=1. Identities with *AtMTP1* are represented by a dot and differences are written in alphabets. Dashes (–) signify deletions. Six transmembrane domains (TMDI to TMDVI) predicted using TMHMM server v. 2.0 [34] are shaded. The histidine rich loop is located between TMDIV and TMDV.

Genomic sequences of all the *A. halleri MTP1* paralogues were obtained from the partial or complete sequencing of BAC clones. Analysis of the complete sequence of BAC clone 7G24 unravelled shared synteny between *A. halleri* and *A. thaliana* genomes in the *A. thaliana* region harbouring the sole *A. thaliana MTP1* gene. This is in correspondence with the mapping results. Detailed sequence analysis revealed that the genetic structure (*i.e.* the gene order) is exactly the same in both species, except that the *A. halleri* genome displays a direct duplication of a 5 kbp region containing an *MTP1* orthologue and a copy of a Retrovirus-related Pol polyprotein from transposon TNT 1-94. The complete sequence of BAC clone 7G24 thus revealed a fifth *AhMTP1* paralogue. The two *MTP1* paralogues arranged in tandem repeat on BAC clone 7G24 were named as *AhMTP1-A1* and *AhMTP1-A2*. They displayed 100% identity in the promoter, 5′ UTR and 3′ UTR regions and differed by only two nucleotides in the coding DNA sequence. One of these differences resulted in an A_365_S substitution and the other one was silent (Figure 3B). The AhMTP1-A2 predicted protein showed 100% sequence identity with the already published full length AhMTP1 predicted protein [14]. The two nucleotidic differences discriminating *AhMTP1*-*A2* from *AhMTP1*-*A1* were shared with *AtMTP1*, *AhMTP1*-*A2* thus being more similar to *AtMTP1* than *AhMTP1*-*A1* (Figure 3).

*AhMTP1-B*, *AhMTP1-C* and *AhMTP1-D* sequences were obtained from BAC clones 12L21, 2B14 and 1F18, respectively. They comprised at least 1.3 kb of putative promoter sequence, the complete 5′ UTR and coding DNA sequences, and at least 156 bp of the putative 3′ UTR and terminator sequences. At the protein level, the five AhMTP1 paralogues displayed on average 97.5% identity with each other (Figure 3B). The most divergent regions were the cytoplasmic N-terminus and the histidine rich loop between transmembrane domains IV and V; this loop has already been proposed to function as a zinc buffering pocket and a sensor of the zinc level at the cytoplasmic surface [18]. The five AhMTP1 paralogues shared only 91–93% identity with their *A. thaliana* and *A. lyrata* orthologues. Together with the result of the phylogenetic analysis of the *MTP1* family in these species (Figure 3A), this suggests that pentaplication of *MTP1* occurred recently in the *A. halleri* lineage. Such a conclusion is similar as the conclusion drawn for the zinc transporting *HMA4* P_1B_type-ATPase, which is present in one copy in *A. thaliana* and is triplicated in *A. halleri* [15].

**Figure 4:**
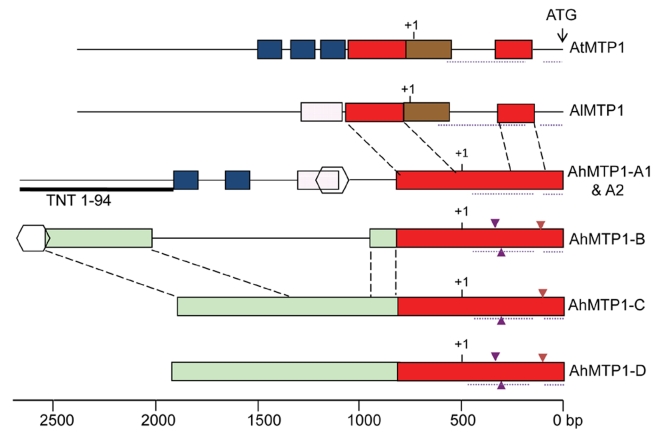
Physical maps comparing the putative promoter plus 5′ UTR regions among *A. thaliana*, *A. lyrata*, and *A. halleri MTP1* homologues. Regions sharing >80% identity are shown by same coloured rectangular boxes or by same shapes. Dashed lines between different gene structures enable the relative positioning of the similar regions. Dotted lines below the gene structures indicate the position of introns. Small triangles present below or above the gene structures indicate 11 bp to 17 bp insertions (see text). Putative transcription start sites and translation start sites are indicated by +1 and ATG respectively. The Retrovirus-related Pol-polyprotein from transposon TNT 1–94 located in the putative promoter region of *MTP1*-*A* is represented by a thick line below the gene structure. Scale is shown at the bottom, relative to the ATG initiation codon.

No intron was present in the region corresponding to the coding sequence for the five *AhMTP1s*, as for *AtMTP1* and *AlMTP1*. In contrast, two introns were present in the 5′ UTR of all *MTP1* orthologues (Figure 4), as revealed by the comparison between the genomic sequences and the published *AhMTP1* and *AtMTP1* mRNA sequences (AJ556183 and AF072858 accession numbers, respectively). Two 11 and 12 bp-long indel differences located in the first intron and a 17 bp-long indel difference located in the second intron discriminated the *AhMTP1* paralogues (triangles in Figure 4). The proximal 800 bp region located upstream of the start codon were on average 95% identical among *AhMTP1s* (red boxes in Figure 4). In contrast, *AhMTP1s* showed on average 65–70% identities with *AtMTP1* or *AlMTP1* in that region. The more upstream parts of the *MTP1* putative promoter regions were markedly divergent (Figure 4). At that location, *AhMTP*-*A1* and -*A2* displayed only few short regions in common with either *AtMTP1*/*AlMTP1* or with the other *AhMTP1s* (blue or pink boxes in Figure 4). The *AhMTP1-C* and *AhMTP1-D* putative promoter regions shared 95% identity over their entire length and on average 97% identities with the *AhMTP1-B* promoter region only on distal sides (green boxes in Figure 4). Scanning of these *MTP1* sequences was performed to identify transcription factor recognition motives that could be important in relation to zinc physiology. The “TGCACAC” conserved motif of metal response element b (MREb) [23] was found in all the *MTP1s* considered here but it was located in the coding DNA sequences. Apart from this MREb motif, no other metal responsive motives were found in the putative promoter regions of any of the *AhMTP1s*. The putative 3′ UTR regions of *AhMTP1-A1*, *-A2*, *-C* and *-D* were found to be 100% identical among themselves, and shared 97% identities with the 3′ UTR regions of either *AtMTP1* or *AlMTP1*. In contrast, *AhMTP1-B* showed only 59% identities with other *MTP1s* in its 3′ UTR region.

Following the principle of parsimony an order of origin of *AhMTP1* duplicates can be hypothesized based on genetic mapping, phylogenetic and sequences analyses. Because phylogenetically *AtMTP1* is more closely related to *AhMTP1*-*A1* and -*A2* than to other *AhMTP1s* and because the *AhMTP1*-*A* locus was mapped to a region that shares conserved synteny with the *A. thaliana* region harbouring *AtMTP1*, it is therefore considered that *AtMTP1* and *AhMTP1*-*A* have been derived from the *MTP1* locus present in the common ancestor of *A. thaliana* and *A. halleri*. Thus, the *AhMTP1*-*A* locus harbours the parent *MTP1* copy of other *A. halleri MTP1s*. Within this locus, it seems impossible to predict whether *AhMTP1*-*A1* or -*A2* is the parent *MTP1* copy as they differ by only two nucleotides. From this parent copy it seems unlikely that *AhMTP1*-*B*, -*C* and -*D* have been independently derived because these three segmental duplicates show higher similarity to each other than to *AhMTP1*-*A* in coding as well as in putative promoter regions. *AhMTP1*-*C* or -*D* probably duplicated from one another because they share >95% identities over their entire length, but it seems impossible to predict which one of them was the first one to come into existence. *AhMTP1*-*B* is the most different from the *AhMTP1-A*. Its 3′ end in particular completely differs from the 3′ ends of other *AhMTP1s* that are 100% identical. Thus, *AhMTP1*-*B* is probably a segmental duplicate of either *AhMTP1*-*C* or -*D*.

### The *AhMTP1*-*D* paralogue is not fixed in the *A. halleri* Auby population

In some experiments, the *AhMTP1*-*D* paralogue could not be found in a few *A. halleri* plants within the Auby population. In order to verify the presence of all the *AhMTP1* gene copies in all the *A. halleri* plants within the Auby population, 188 plants were selected from this population and PCR was done using gene copy specific primer pairs (Table S1). The 188 plants were collected every ∼3 m along a 500 m-long transect starting from the least polluted zone at the periphery of the site towards the most polluted zone close to the centre of the site. This choice was made to check whether there could be a link between the possible presence of *AhMTP1*-*D* and zinc concentration in soil. In order to overcome possible allelic variation interfering with our analysis, two independent primer pairs enabling the specific detection of *AhMTP1*-*D* were designed. This strategy was chosen so that a lack of amplification by both primer pairs ascertains absence of the *AhMTP1*-*D* copy.

**Figure 5:**
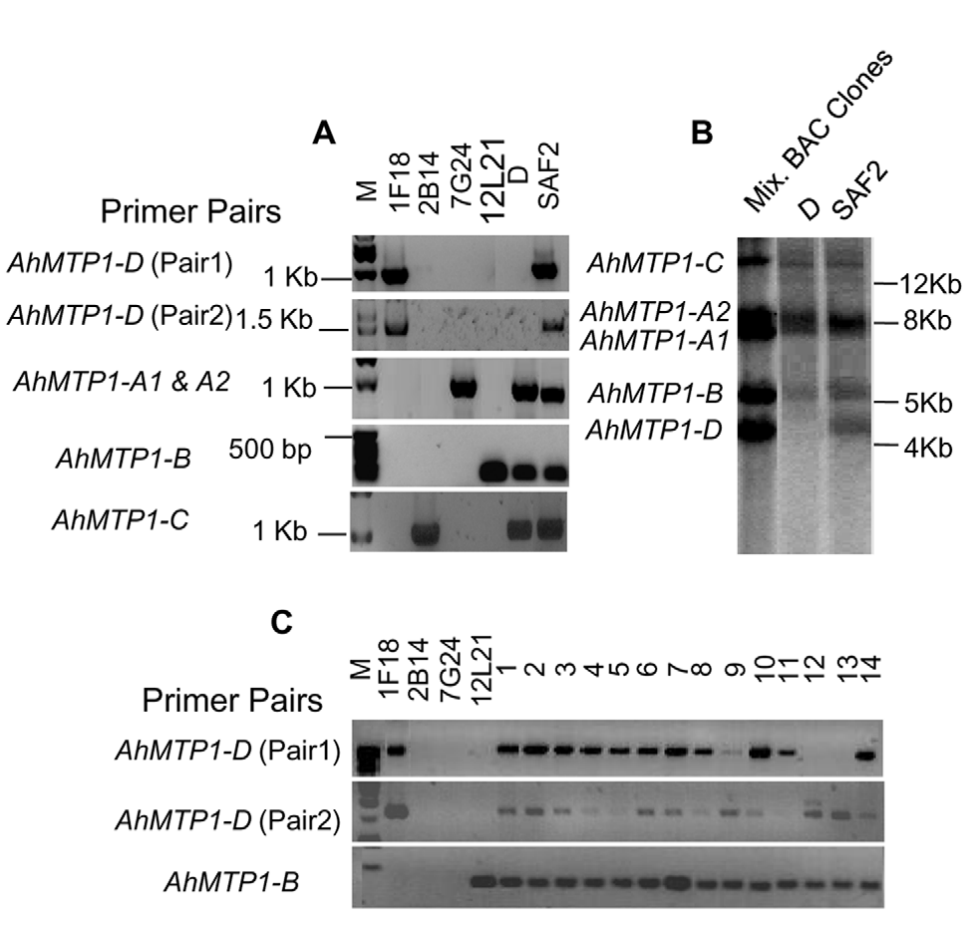
Analysis of the presence of the *MTP1-D* paralogue in different *A. halleri* genotypes. (A) PCR analysis of two plants of the *A. halleri* Auby accession (D and SAF2 plants) using *AhMTP1*-*A*, -*B*, -*C*, and -*D* gene specific primer pairs. DNA from BAC clones 1F18, 2B14, 7G24 and 12L21 are positive controls for demonstrating the specific amplification of *AhMTP1*-*D*, -*C*, -*A*, and -*B,* respectively. Corresponding sizes of amplicons are indicated on the right of each lane. (B) Southern analysis of the D and SAF2 plants of the Auby accession. Plant genomic DNAs and mixed BAC clone DNAs (positive control) were digested with *Pst*I restriction enzyme. The probe was amplified from the *AhMTP1-D* harbouring BAC clone. Sizes deduced from a ladder are shown to right of the panel. (C) Production of *AhMTP1-D* specific amplicons from plants belonging to 14 different *A. halleri* populations. The 1 through 14 numbers above the lanes represent the following *A. halleri* populations, respectively: M Auby, M Sauerland, NM Regen, M Harz, NM CZ8-13, NM nord Tyrol, M Katowice-Weinowice, NM Zakopane, NM Appusenes, NM Fagaras Ro-12-6, NM Fagaras Ro-ovirensis, NM Southern Tyrol, M Lombardie and NM Tessin, where M = metallicolous and NM = non metallicolous. *AhMTP1-B* specific primer pairs were used as a control.

*AhMTP1*-*A1* & *A2*, -*B* and -*C* amplicons were produced from all the plants (Figure S1 and data not shown). In contrast, 25% of the plants produced no amplicon for *AhMTP1*-*D*. This indicated that the *AhMTP1*-*D* gene is not fixed in the Auby population. No correlation was observed between the ability of a plant to produce *AhMTP1*-*D* amplicons and its position in the transect (data not shown). In order to ascertain that the *AhMTP1*-*D* paralogue was missing in some of the plants from the Auby accession, a Southern analysis was performed to analyse a plant producing no amplicon with *AhMTP1*-*D* specific primer pairs (D line) and another plant producing amplicons (SAF2 line). Comparison between the hybridization profiles of both plants and of a mix of the *AhMTP1s* harbouring BAC clones revealed that the bands specific to *AhMTP1*-*D* was only detected in the SAF2 line while the bands corresponding to *AhMTP1*-*A1*, -*A2*, -*B* and -*C* were detected in both lines (Figure 5A and 5B). These results confirmed that *AhMTP1*-*D* is not fixed in the Auby population.

Presence of the *AhMTP1-D* copy was assayed by PCR in 14 different *A. halleri* populations representing the whole geographic distribution of the *A. halleri* species (Figure 5C). Interestingly at least one of the *AhMTP1-D* specific primer pairs produced amplicon for all the plants representing these populations. This indicates that the *AhMTP1-D* copy is present in all the analysed accessions. Our analysis cannot help to determine whether *AhMTP1-D* is fixed or not in all the *A. halleri* accessions. However, it can be concluded that *AhMTP1-D* is not in the process of being gained specifically in the *A. halleri* Auby population.

### The *AhMTP1* paralogues differentially complemented the zinc hypersensitivity of the *zrc1 cot1* yeast mutant

**Figure 6:**
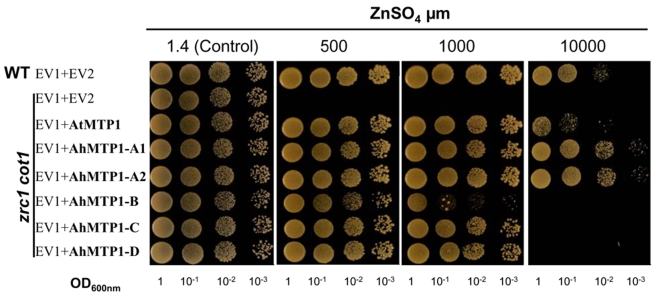
Functional complementation of the zinc-hypersensitivity of the *zrc1 cot1* yeast mutant by *AhMTP1*s and *AtMTP1*. Wild-type BY4741 (WT) and mutant *zrc1 cot1* yeast strains harboured the empty pFL38H (EV1) that brought histidine autotrophy and either empty pYX212 vector (EV2) or pYX212 bearing one of *MTP1s*. Serial dilutions were spotted on modified selective LSP medium supplemented with different concentrations of ZnSO_4_ as indicated above the panels. Each spot was made with 10 µl of a yeast culture diluted at the OD_600nm_ mentioned below the drops. Pictures were taken after 2 days for control and 4 days for other treatments.

The five AhMTP1s and AtMTP1 were assayed for their ability to complement the zinc hypersensitivity of the yeast *zrc1 cot1* double mutant, which is defective in vacuolar zinc transport [24]. Drop tests conducted on modified LSP medium supplemented with 500 µM zinc showed that the five AhMTP1s indeed induced functional complementation of the double *zrc1 cot1* mutations (Figure 6). However, increasing zinc concentration in the medium up to 10 mM revealed the differential ability of the paralogues to complement zinc hypersensitivity of the *zrc1 cot1* strain. AhMTP1-A1 and -A2 showed equal and highest complementation. They were slightly more efficient than AtMTP1. In contrast, AhMTP1-B was the least efficient among all of AhMTP1s in imparting complementation. Complementation imparted by AhMTP1-C and -D was equal and intermediate. Similar results were reproduced using independent clones and different media.

### *AhMTP1* transcripts are differentially accumulated *in planta*

**Figure 7:**
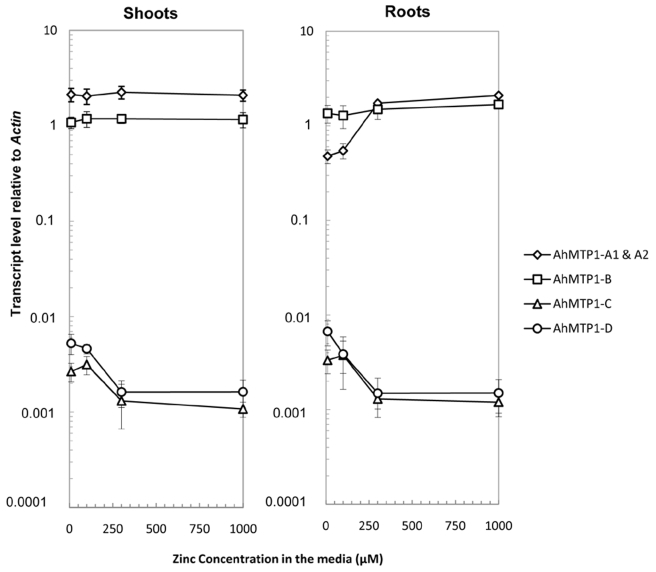
*AhMTP1s* transcript accumulation in plants submitted to different zinc treatments. Roots and shoots were collected from plants of the *A. halleri* SAF2 genotype issued from the Auby accession that were exposed to 10 (control), 100, 300, or 1000 µM ZnSO_4_ for 4 days. Real-time quantitative RT–PCR was performed using gene copy specific primer pairs separately for shoots and roots. Data shown are transcript levels of *AhMTP1s* relative to *Actin*. Each data point in the graph is the average of three PCR repetitions for each of six biological replicates. Errors bars correspond to confidence intervals at the 0.05 threshold.

The transcript accumulation of the different *AhMTP1* paralogues was analysed using Real-time quantitative RT-PCR in shoots and roots of individual mature plants grown in hydroponics on media supplemented with 10 (control), 100, 300 or 1000 µM ZnSO_4_ for 4 days (Table S2). The *AhMTP1-A1* and *-A2* genes sharing 99.9% identities in the whole gene including the promoter region could not be discriminated in that analysis. Otherwise, paralogue-specific primer pairs enabled to discriminate the *AhMTP1-A*, *-B*, *-C* and *-D* genes. In both roots and shoots, *AhMTP1-A1* & *-A2* and *AhMTP1-B* transcripts were much more abundant than *AhMTP1-C* and *AhMTP1-D* ones (Figure 7). On average, the relative abundances differed by nearly three orders of magnitude. In shoots steady-state *AhMTP1-A1* & *-A2* transcripts were more abundant than steady-state *AhMTP1*-*B* transcripts whereas it was the reverse in roots. In response to increasing zinc concentration in the culture medium, *AhMTP1*-*A1* & -*A2* transcripts as well as *AhMTP1*-*B* ones remained stable in shoots. *AhMTP1*-*A1* & -*A2* transcripts were induced in roots while *AhMTP1*-*B* ones remained stable (Figure 7). *AhMTP1-C* and *AhMTP1-D* transcript levels, which were very close to each other, decreased in shoots as well as in roots in response to increasing zinc concentration in the culture medium.

## Discussion

With the aim to better understand the adaptive evolutionary processes leading to zinc hyperaccumulation and tolerance in *A. halleri*, we characterised the *AhMTP1* gene family, which had been proposed to play a role in the control of these traits [14], [17]. Whereas, previous work identified three genetically unlinked *MTP1* loci in *A. halleri*, the present study revealed the existence of five *AhMTP1* paralogues located at 4 different loci. We consider that all the possible *AhMTP1* paralogues have now been identified for the following two reasons. First, the paralogues were obtained from the screening of an *A. halleri* BAC library representing ∼4 equivalent genomes, meaning that any given *A. halleri* gene has a 0.986 probability to be present in at least one BAC clone of the library [21]. Then, the *MTP1* hybridization profiles were identical between DNA mix of our BAC clones and different plant genomic DNAs coming from various accessions (Figure 5B, and compare Figure 1A of this work to Figure 4(a) from [16]).

From the genomic sequences that we obtained, the mechanism(s) responsible for the generation of *MTP1* duplicates could not be identified. However, we could propose the most likely order of origin of theses duplications. Either of *AhMTP1*-*A1* or -*A2* is considered as the parent copy from which either of *AhMTP1*-*C* or -*D* was derived. *AhMTP1*-*B* would then have been derived from either of *AhMTP1*-*C* or -*D*.

Other genes involved in zinc tolerance or in zinc homeostasis display different degrees of multiplication in *A. halleri* as compared to in *A. thaliana*. These are for instance *ZIP3*, *ZIP6* and *ZIP9*, members of the zinc-regulated transporter/iron-regulated transporter-like proteins family [16], the *HMA4* transporter controlling root to shoot zinc transport [15] and type I defensins involved in cellular zinc tolerance [25]. The hypothesis that having more copies of genes involved in zinc homeostasis would be a general characteristic of *A. halleri* can however not be raised since other genes related to zinc tolerance or zinc homeostasis such as *ZIP10*, *IRT3* or *FRD3* are present as single copies in *A. halleri* as in *A. thaliana* [16]. In this context, the fact that five *MTP1* copies are present in *A. halleri* may be the consequence of the fact that *MTP1* plays a critical function with respect to zinc detoxification. This led us to study functional characteristics of the five paralogues.

The five *AhMPT1* paralogues displayed a significant diversity in their ability to functionally complement the zinc hypersensitivity of the *S. cerevisiae zrc1 cot1* mutant (Figure 6). While different causes can underlie this diversity, we favour the hypothesis that the differential functionality of the AhMTP1 paralogous proteins is linked to amino acid sequence differences. AhMTP1 predicted protein sequences displayed the greatest density of differences in a histidine rich loop located between transmembrane domains IV and V that has already been proposed to be a main regulatory domain of the protein [18]. Although it cannot be excluded that amino acid differences in other domains of the protein may also explain the functional differences between AhMTP1s, it seems likely that differences within the histidine rich loop are the determining factors for the differential ability of the AhMTP1s to complement the zinc hypersensitive phenotype of the mutant yeast.

The greatest functional difference discriminating the five *AhMTP1* paralogues relates to their transcript levels, which varied by nearly three orders of magnitude (Figure 7). Our results are a bit different from previous ones [14] concerning the relative transcript abundances of the different *AhMTP1* paralogues. We consider our data to be more accurate since we performed quantitative PCR and used gene specific probes made from clearly identified clones, which was not the case in the previous study. Alternatively, differences between our findings and previous ones might be due to different growing conditions in the two studies. One interesting novelty is that two of the *AhMTP1* paralogues, *AhMTP1-C* and *-D*, displayed reduction in mRNA abundances following application of zinc. These paralogues were also the ones that showed a markedly lower transcript abundance compared to the three other ones in normal growing condition. This might be the sign that *AhMTP1-C* and *-D* play a completely different function from the other paralogues. Unsurprisingly, the differences between the transcript levels of the five *AhMTP1* paralogues are in coherence with the differences between the corresponding promoter sequences (Figure 4 and Figure 7). For instance, the *AhMPT1-D* paralogue was found to display a transcript accumulation profile very similar to that of *AhMTP1-C*. This similarity can be related to the 95% identities observed between the putative promoter regions of the two paralogues. The extensive analysis of the relationship between *AhMTP1* transcript levels and promoter sequences lead us to specific regions that may be responsible for the control of either the level of expression or the response to the zinc constraint. Among these, the 400 and 1000 bp long regions differentiating the putative *AhMTP1*-*B* promoter and the putative *AhMTP1*-*C* promoter should bring the most fruitful outcomes.

Among the genes proposed to be involved in zinc tolerance in *A. halleri*, only *IRT3* and *HMA4* have been fully characterised at both the genomic and functional levels [15], [26]. The situations characterising *MTP1*, *IRT3* and *HMA4* in *A. halleri* are completely different. First, *AhIRT3* is in single copy while *AhHMA4* is triplicated, with the three paralogues being present in tandem, and *AhMTP1* is pentaplicated with both tandem and segmental duplicates. Transcripts of all three gene families were over-accumulated in *A. halleri* as compared to in *A. thaliana* [14], [15], [26]. However, analysing the relative contribution of different gene copies revealed completely different situations. For *IRT3*, transcript over-accumulation was attributable to the sole copy [26] while for *HMA4*, it was mainly due to the additive and equal contribution of the three paralogues [15]. In contrast, transcript over-accumulation could only be attributed to three of the five members of the *AhMTP1* family (this work). The *MTP1* family thus displays original characteristics in *A. halleri*. Remarkably, these original characteristics are not observed in another zinc hypertolerant and hyperaccumulating species, *Thlaspi goesingense*, as this species harbours only one *MTP1* copy, which is over-expressed compared to in *A. thaliana* [27]. These characteristics displayed by *AhMTP1* duplicates suggest that different evolutionary fates might take place for the duplicates in *A. halleri*.

As mentioned above, gene duplication together with mutations occurring in duplicates are promoting novelty in the evolutionary process [1]. Then, genes can be exposed to different kinds of evolutionary fates: sub-functionalization, neo-functionalization or non-functionalization [7]. Analysing the *AhMTP1* gene family revealed asymmetric relationships among the *AhMTP1* duplicates from the point of view of transcript accumulation patterns and protein function. Transcripts of *AhMTP1-A1*, *-A2* and *-B* were found to be far more abundant than transcripts of *AhMTP1-C* and *-D*. At the same time, *AhMTP1-B* was less competent than *AhMTP1-C* and *-D* in complementing the zinc-hypersensitivity of the *S. cerevisiae zrc1 cot1* double mutant. Since *AhMTP1-A1*, *-A2* and *-B* were found to co-localize with previously described zinc-tolerance QTLs for short term root elongation whereas *AhMTP1-C* and *-D* did not, it appears that transcript abundance is a more important factor controlling the contribution of *AhMTP1s* to zinc tolerance in *A. halleri* than the ability of protein itself to confer zinc tolerance. In this context, the fate of *AhMTP1s* duplications could be sub-functionalization for the *AhMTP1*-*A1*, -*A2* and -*B* copies. This hypothesis needs to be validated by systematic comparison of single, double and triple mutants for each gene copy to assess the functional redundancy of these paralogues. By contrast, the *AhMTP1*-*C* and -*D* duplicates are more difficult to characterise. Because *AhMTP1*-*C* and -*D* are neither expressed well nor present in the previously described zinc-tolerance QTLs, they may appear to be in the process of non-functionalization. This hypothesis would be supported by the additional observations that *AhMTP1*-*D* is not fixed in the metallicolous *A. halleri* Auby population, and that its occurrence in plants from this accession is unlinked to the zinc concentration in the soil, which reveals freedom from selective pressure. However, since the AhMTP1-C and -D proteins functionally complemented the yeast mutant and the corresponding genes are both still expressed *in planta*, another hypothesis can be raised. This hypothesis would be that changes in the regulatory profile of these copies would correspond to a neo-functionalization process [28] leading *AhMTP1*-*C* and -*D* to play another role than being involved in zinc tolerance for short term root elongation. In that situation, the interpretation of the non fixed nature of the *AhMTP1*-*D* in the Auby population could be that this copy is redundant with *AhMTP1*-*C*. Concluding on the actual evolutionary fate of the *AhMTP1*-*C* and -*D* copies would thus require an extensive analysis of the temporal and spatial expression patterns of all the *AhMTP1* paralogues. Fates of duplicates were proposed to differ depending on the nature of the duplication [6]. Tandem duplication was proposed to provide a means of amplifying adaptatively important genes, particularly resistance genes, while segmental duplication was proposed to permit gene family diversification and long-term evolutionary plasticity. Our findings might be in agreement with this assumption. Indeed, the *AhMTP1*-*A1* & -*A2* tandem duplicates experience sub-functionalization, while two of the three segmental duplicates experience either neo- or non-functionalization.

In conclusion, based on genomics as well as functional approaches we propose that different evolutionary fates are likely to take place for *AhMTP1* duplicates. Two paralogues do not appear to be under selective pressure for zinc tolerance, while three others appear to be. This study thus brings important outcomes to understand the mechanisms underlying the adaptation of *A. halleri* to zinc.

## Materials and Methods

### Plant material

Two different micropropagated lines from the *A. halleri* Auby population (the D and SAF2 lines) were used for Southern hybridization and/or transcript accumulation analyses. For the analysis of the presence of all the *MTP1* paralogues in accessions representing the genetic diversity within the *A. halleri* species, one plant was taken at random from each of the following populations: M Auby from France, M Sauerland, NM Bavarian Forest and M Harz from Germany, NM CZ8-13 from Czech Republic, NM Nord Tyrol from Austria, M Katowice-Weinowice and NM Zakopane from Poland, NM Apuseni mountains, NM Fagaras Ro-12-6 and NM Fagaras Ro-ovirensis from Romania, NM Southern Tyrol, M Lombardie and NM Tessin from Italy, where M qualifies a metallicolous population and NM a non metallicolous one according to already described criteria [13].

### BAC clone handling and Southern hybridisation

BAC clone identification was performed through the Southern screening of a BAC library made from an *A. halleri* plant from the Auby population, as described [21]. Then, BAC clone DNA was extracted from 3 ml of overnight culture grown in 2YT medium containing 12.5 mg/ml chloramphenicol, using the Nucleobond Plasmid DNA Purification kit (Macherey Nagel) but skipping the column purification step. *AhMTP1-A1* and *A2* sequences were obtained by full length sequencing of the 7G24 BAC clone (Genoscope, Evry, France). *AhMTP1-B*, *-C* and *-D* genomic sequences were obtained by partial sequencing of BAC clones 12L21, 2B14 and 1F18, respectively (GATC Biotech, Konstanz, Germany and Genoscreen, Lille, France). Sequences were deposited in the EMBL database. Accession numbers of the 7G24 BAC clone sequence and of the *AhMTP1-B*, *-C* and *-D* genomic sequences are (FN428855), (FN386317), (FN386316), and (FN386315), respectively. Sequences from which BAC clone specific genetic markers were designed are available under the accession numbers (FN386313) for BAC clone 2B14, (FN386314) and (FN428827) for BAC clone 1F18, (FN386317) for BAC clone 12L21 and (FN428855) for BAC clone 7G24.

For Southern analyses, 2 µg of BAC clone DNA or 10 µg of *A. halleri* genomic DNA were digested with 50 U of restriction enzyme at 37°C for 6–7 h and separated on a 0.8% (w/v) agarose gel in TEA1X buffer. Then, the agarose gel was submerged into 0.25 N HCl for 15 min and rinsed with water 2–3 times. DNA fragments were transferred onto a positively charged nylon membrane (Hybond-N+, Amersham Biosciences) by capillary action using 0.4 N NaOH for 8 h and then cross-linked onto the membrane for 80 sec under 254 nm UV light at 0.120 J.cm^−2^ with the Fluo-Link apparatus (Bioblock, Illkirch, France). For Southern analysis of BAC clones, the *MTP1* probe was obtained from a PCR fragment produced from *A. halleri* genomic DNA using the 5′ -CGAGTCTTCAATTTCTGCAACT-3′ and 5′-AAACTTTATTGATTTATTGTTAA-3′ primers and purified using the Wizard SV Gel and PCR clean-up system (Promega). For the Southern analysis of genomic DNA, the probe was obtained from a PCR fragment produced from the 1F18 BAC clone using the 5′-TTTTCGGTTAAAGCGCACG-3′ and 5′-TGCAGAACTCGAAATCAAC-3′ primers. Fifty nanograms of purified PCR product were radioactively labelled by random priming (Prime-a-gene kit, Promega). The probe was then purified on illustra NICK columns (GE Healthcare). Prehybridization was carried out in Church buffer [29] for >2 h. Hybridization was carried out in the same buffer overnight at 65°C. Then the blots were washed. The final and more stringent wash was 0.5XSSC, 0.1% (w/v) SDS for BAC clones Southern blots and 0.1XSSC, 0.1% (w/v) SDS for genomic Southern blots for 20 min at 50°C. Blots were then placed against "Imaging Plate BAS-MS" screens, which were revealed using a BAS 5000 apparatus (Fujifilm, Japan).

### Mapping of *AhMTP1* paralogues on the *A. halleri* X *A. lyrata petraea* BC1 genetic map

Already available genomic DNA of the parents of the *A. halleri* X *A. lyrata petraea* BC1 population and of 199 plants from this population was used for genotyping, as described [30]. Mapping of the 7G24, 12L21, 2B14 and 1F18 BAC clones harbouring *MTP1-A*, *-B*, *-C* and *-D*, respectively, was performed by CAPS and SSCP analysis as described [20], using the markers described in Table S3. To make these markers, primer pairs were designed from sequences of the BAC clones and tested on genomic DNA of the *A. halleri* and *A. lyrata petraea* parents of the BC1 population. When no SSCP polymorphism could be detected, CAPS-type markers were made, as a result of assaying a set of different restriction enzymes on *A. halleri* and *A. lyrata petraea* amplicons.

Genotypes obtained in the BC1 population for the BAC-derived markers were combined with the data set used for the *A. halleri* X *A. lyrata petraea* linkage map construction [20], [30], using the Joinmap 3.0 program [31]. Individuals lacking information for more than 25% of all markers were excluded from the analysis. Linkage groups were obtained at a logarithm-of-odds (LOD) score threshold of 4. The best order of markers along each linkage group was determined using the sequential method implemented in Joinmap, comparing the goodness-of fit of the resulting map for each tested order using thresholds of 0.5 and 1.0 for the linkage groups and the loci, respectively. Translating recombination frequencies into map distances was made using Kosambi's mapping function [32].

### Functional complementation in yeast

*A. halleri* and *A. thaliana MTP1s* are without any intron in the region corresponding to the coding DNA sequence. Thus their full length open reading frames were amplified directly from genomic or BAC DNAs using the proofreading *Pfu* DNA polymerase (Promega). *A. halleri MTP1s* were amplified using the forward 5′-AAAGAATTCATGGAGTCTTCAAGTCAC-3′ and reverse 5′-CCCCTCGAGTTAACGCTCGATTTGTATCG-3′ primers containing *Eco*RI and *Xho*I restriction sites, respectively (underlined sequences). *A. thaliana MTP1* was amplified using the forward 5′-AAAGAATTCATGGAGTCTTCAAGTCCCCAC-3′ and reverse 5′-CCCCTCGAGTTAGCGCTCGATTTGTATCG-3′ primers also containing *Eco*RI and *Xho*I restriction sites, respectively. The PCR products were cloned downstream of the triose phosphate isomerase promoter in the pYX212 yeast expression vector at the *Eco*RI and *Xho*I restriction sites. A *Saccharomyces cerevisiae zrc1 cot1* mutant (*Mat a*, *zrc1::natMX3*, *cot1::kan-MX4*, *his3Δ1*, *leu2Δ0*, *met15Δ0*, *ura3Δ0*) and its parental wild-type strain BY4741 were double transformed with the empty pFL38H (his+) vector and either empty pYX212 (ura+) or pYX212 (ura+) expressing *AhMTP1-A1*, *-A2*, *-B*, *-C*, *-D* or *AtMTP1*, using the lithium acetate/single-stranded carrier DNA/polyethylene glycol method [33]. Systematic co-transformation with pFL38H empty vector was done to avoid addition of histidine in the culture medium, as histidine is supposed to be a zinc chelator in growth medium. For drop assays, transformed yeast strains were grown overnight in 5 ml selective liquid YNB medium to early stationary phase. Yeast cells were then washed twice with ultrapure H_2_O and diluted in water to OD_600nm_ = 1, 0.1, 0.01 and 0.001. Drop assays were performed on selective modified Low Sulphate/Phosphate medium [14] with pH adjusted to 4.7. The medium was supplemented with various concentrations of ZnSO_4_: 1.4 µM for control condition and from 100 µM to 10 mM for zinc treatments. At least four independent colonies were tested for each construct.

### Gene expression analysis

For Real-Time Quantitative RT-PCR experiments, the *A. halleri* SAF2 genotype from the Auby accession was micropropagated *in vitro* on standard Murashige and Skoog culture medium with 0.8% (w/v) sucrose and 2% (w/v) agar. Four weeks later, rooted clones were transferred in hydroponics as described [25] and were let to acclimate themselves to this medium for 6 days. Then, individual clones were submitted for 4 days to different zinc treatments: 10 µM (control), 100 µM, 300 µM and 1000 µM ZnSO_4_. Six replicates were treated in parallel for each condition and analysed independently. Roots and shoots were harvested separately.

Total RNA was extracted (RNeasy kit; Qiagen, Hilden, Germany) then genomic DNA was removed using the RQ1 RNase-Free DNase kit (Promega). Four micrograms of total RNA were used as a template for first strand cDNA synthesis, which was performed using M-MLV Reverse Transcriptase, RNase H Minus, Point Mutant (Promega) and Oligo (dT)_15_ Primer (Promega) in a final volume of 100 µl, according to the instructions from the manufacturer.

Real-time RT-PCRs were performed in 384-well plates with the LightCycler 480 Real-Time PCR System (Roche diagnostics GmbH, USA) using SYBR Green to monitor cDNA amplification. Two microlitres of DNA sample were then used for PCR in a 10 µl reaction mixture containing 5 µl of LightCycler 480 SYBR Green I Master kit (Roche diagnostics GmbH) and 0.5 µM of each primer. The primer pairs used for the transcript accumulation analysis were designed in the specific regions of different *AhMTP1s* (Table 1). *Actin* was considered as an internal control. The primers used to analyse *Actin* have already been described [25]. The PCR program started with an initial 5 min-long treatment at 95°C. Then the samples were submitted to 45 PCR cycles composed of 10 sec at 95°C, 10 sec at respective annealing temperatures for each primer pair (Table 1) and 10 sec at 72°C. The specificity of the amplified PCR products was assessed for every sample by analysing the amplicon dissociation during the gradual increase of the temperature from 72°C to 95°C at the rate of 0.11°C/sec, using the Tm calling method proposed by the LightCycler 480 Software release 1.5.0. Six out of 750 PCR reactions showed unspecific amplification. The corresponding data were discarded.

**Table 1:** Gene-specific primer pairs used to characterise the different *AhMTP1* paralogues in real-time quantitative RT–PCR analyses.

Gene	Primer name	Primer sequence 5′--3′	Annealing temperature (°C)	Average PCR efficiencies	
				gDNA	cDNA
*Actin*	ActinF	GGTAACATTGTGCTCAGTGGTGG	65.9	1.87	1.90
	ActinR	AACGACCTTAATCTTCATGCTGC			
*AhMTP1-A1 &A2*	ARTF	AGTGGTGAACATCATAATGGCTGTTC	67.2	1.96	1.93
	ACRTR	TCGATTTGTCCAACAGTTGCTCAG			
*AhMTP1-B*	BRTF	TGGACGTGAAGTTATGGAGAC	64.3	2.06	2.04
	BRTR	CTTCACATTTGGGCTATCACAG			
*AhMTP1-C*	CRTF	GGTGAACATCATAATGGCTGTTATGCTG	67.8	1.98	NP a
	ACRTR	TCGATTTGTCCAACAGTTGCTCAG			
*AhMTP1-D*	DRTF	GTACACCCAGAGAGATTGACGCCG	71.3	1.91	NP a
	DRTR	GACTAATGTTGTACTCCCTGCGGATG			

**a** NP: Not possible to be done (see Materials and Methods).

For each primer pair specific to *AhMTP1* paralogues, the PCR efficiency (E) was determined after the analysis of 5 serial 1∶10 dilutions of BAC clone DNA (Table 1) by using the equation E = (10^−1/s^), where “s” is the slope of the linear regression of the threshold cycle (Ct) values per the log_10_ values of the starting DNA copy numbers. When analysing the cDNA samples, the PCR efficiency was also evaluated from the analysis of 1∶3, 1∶12 and 1;48 dilutions of first strand cDNA (Table 1, Table S2) for *Actin*, *AhMTP1*-*A1* and -*A2* and *AhMTP1*-*B*. Transcript accumulation of the different genes was calculated using the respective experimentally determined PCR efficiency values for each primer pair. For the *AhMTP1*-*C* and -*D* genes, results from the 1∶12 and 1∶48 cDNA dilutions were unreliable because of surpassing the trustworthy detection limit of the real-time quantitative RT-PCR and were thus discarded (Table S2). For the *AhMTP1*-*C* and -*D* genes, it was thus impossible to calculate PCR efficiencies on cDNA samples; efficiencies measured on BAC clone DNA were thus used for calculations. Relative expression levels (REL) of *AhMTP1s* compared to the *Actin* were determined for every sample from the result of the 1∶3 dilution using the equation REL  =  [(E)^-Ct^]*_AhMTP1_* / [(E)^-Ct^]*_Actin_*, where E and Ct are the PCR amplification efficiency and the threshold cycle, respectively, for the considered *AhMTP1* and *Actin*. Six independent plant samples were considered for each condition.

## Supporting Information

Figure S1 Analysis of the presence of the *AhMTP1* paralogues in 44 plants from the Auby accession using gene copy specific primer pairs. Each of the horizontal panel show the amplification obtained from a primer pair specific to the *MTP1* paralogue named at the left of the panel. Samples from BAC clones 1F18, 2B14, 7G24, and 12L21 were used as controls for specificity of the primer pairs. The lane M represents1kb invitrogen DNA ladder.
(0.09 MB PDF)

Table S1 Sequences of gene-specific primer pairs and the corresponding annealing temperatures.
(0.03 MB DOC)

Table S2 Threshold cycles (Ct) obtained in quantitative RT-PCR analyses performed on cDNAs coming from shoots and roots of *A. halleri* plants.
(0.06 MB XLS)

Table S3 Markers used for genetic mapping of *AhMTP1* harbouring BAC Clones.
(0.04 MB DOC)
