# Supplementary material for: The Five AhMTP1 Zinc Transporters Undergo Different Evolutionary Fates towards Adaptive Evolution to Zinc Tolerance in Arabidopsis halleri
Source: PLoS Genet. 2010 Apr 15;6(4):e1000911. doi: 10.1371/journal.pgen.1000911 (PMC2855318; doi:10.1371/journal.pgen.1000911)
Supplement: Figure S1 — Analysis of the presence of the AhMTP1 paralogues in 44 plants from the Auby accession using gene copy specific primer pairs. Each of the horizontal panel show the amplification obtained from a primer pair specific to the MTP1 paralogue named at the left of the panel. Samples from BAC clones 1F18, 2B14, 7G24, and 12L21 were used as controls for specificity of the primer pairs. The lane M represents1kb invitrogen DNA ladder. (0.09 MB PDF) [file pgen.1000911.s001.pdf]

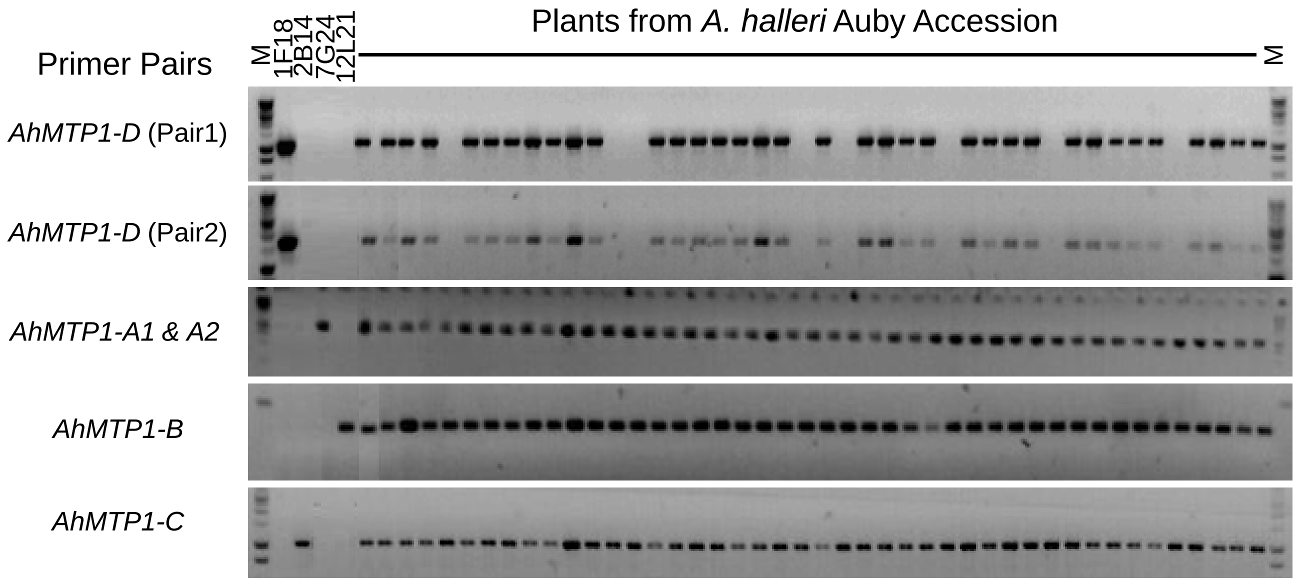

Figure S1; Analysis of the presence of the *AhMTP1* paralogues in 44 plants from the Auby accession using gene copy specific primer pairs. Each of the horizontal panel show the amplification obtained from a primer pair specific to the *MTP1* paralogue named at the left of the panel. Samples from BAC clones 1F18, 2B14, 7G24 and 12L21 were used as controls for specificity of the primer pairs. The lane M represents1kb invitrogen DNA ladder.
