# Supplementary material for: The Five AhMTP1 Zinc Transporters Undergo Different Evolutionary Fates towards Adaptive Evolution to Zinc Tolerance in Arabidopsis halleri
Source: PLoS Genet. 2010 Apr 15;6(4):e1000911. doi: 10.1371/journal.pgen.1000911 (PMC2855318; doi:10.1371/journal.pgen.1000911)
Supplement: Table S1 — Sequences of gene-specific primer pairs and the corresponding annealing temperatures. (0.03 MB DOC) [file pgen.1000911.s002.doc]

**Table S1; Sequences of gene-specific primer pairs and the corresponding annealing temperatures**

| Gene | Primer Name | Primer Sequence 5/--3/ | Annealing temperature (°C) |
| --- | --- | --- | --- |
| *AhMTP1-A* | 7G24-ge3 | CTGTGGAGAAGCGCCTTGTGA | 68.4 |
|  | 7G24-ge4 | GCAGACCCTTTTCGAGCTTTGT |  |
| *AhMTP1-B* | 12L21-1 | GATGTAGCATCGTCTCCATAAC | 63 |
|  | 12L21-ge4 | CTAAGCTTAAAAGCCACGCAAG |  |
| *AhMTP1-C* | 2B14-ge3 | ATTGTGGGCTGCTAGCTGGGAAGCG | 64.2 |
|  | 2B14-ge4 | CCAAACGATGATGACACATAACTAT |  |
| *AhMTP1-D* (Pair 1) | 1F18-1 | TAGCTTTCTTTTGGCCTTGTCC | 60 |
|  | MTP1-3D | AACAGCCATTATGATGTTCACCACTAG |  |
| *AhMTP1-D* (Pair 2) | 1F18-ge5 | CTTAGAATTGGTAACGCTC | 58 |
|  | 1F18-ge6 | GCTTACTTGTGCTCAACCAAAC |  |
