# Supplementary material for: The Five AhMTP1 Zinc Transporters Undergo Different Evolutionary Fates towards Adaptive Evolution to Zinc Tolerance in Arabidopsis halleri
Source: PLoS Genet. 2010 Apr 15;6(4):e1000911. doi: 10.1371/journal.pgen.1000911 (PMC2855318; doi:10.1371/journal.pgen.1000911)
Supplement: Table S3 — Markers used for genetic mapping of AhMTP1 harbouring BAC Clones. (0.04 MB DOC) [file pgen.1000911.s004.doc]

**Table S3; Markers used for genetic mapping of *AhMTP1* harbouring BAC Clones**

| Marker | Primer Name | Primer Sequence 5/--3/ | Marker Type | Enzyme Used | Fragment sizes (bp) | |
| --- | --- | --- | --- | --- | --- | --- |
|  |  |  |  |  | A. halleri | A. lyrata |
| 7G24 | MTP1-1 | TTCTGGTGAGCCCTTCTTCCTC | SSLP |  | 186 | 300 |
|  | MTP1-2 | CTCGTCAAGCAAACAAGCCAGTGTC |  |  |  |  |
| 12L21 | 12L21-2 | GAATCTCTTGAGACAGACTTCC | PCR-dominant |  | 1086 | No amplification |
|  | 12L21-3 | TGCTCACGGGTATTCTGGTTTATG |  |  |  |  |
| 2B14 | 2B14-fbgm1 | CTGTTTGCTGCCTACATATATTAG | CAPS | EcoRV | 221, 335 | 525 |
|  | 2B14-fbgm2 | GAATATCGGGAAGAAATGGTG |  |  |  |  |
| 1F18for | 1F18-fbgm1 | TCTAGAGATGGAGCTCG | CAPS | TaqI | 101, 206, 209 | 5, 42, 46, 367 |
|  | 1F18-fbgm2 | CTAGGCTAACTAGAGTTCCGA |  |  |  |  |
| 1F18rev | 1F18rbgmF2 | GATAATAGTACCTAACGCCGTC | CAPS | TaqI | 40, 75, 100, 360 | 40, 75, 100, 160, 200 |
|  | 1F18rbgmR2 | CGACCATTTCTATTTCCCCTAATG |  |  |  |  |
